# Clinical Follow-Up of Responses to Treatment with Benznidazol in Amazon: A Cohort Study of Acute Chagas Disease

**DOI:** 10.1371/journal.pone.0064450

**Published:** 2013-05-27

**Authors:** Ana Yecê das Neves Pinto, Vera da Costa Valente, José Rodrigues Coura, Sebastião Aldo da Silva Valente, Angela Cristina Veríssimo Junqueira, Laura Cristina Santos, Alberto Gomes Ferreira, Roberto Cavalleiro de Macedo

**Affiliations:** 1 Clinical epidemiologic Department of Evandro Chagas Institute (SOAMU-IEC)- Secretaria de Vigilância em Saúde/Brazil Ministery Health (SVS/MS), Belém, Pará, Brazil; 2 Parasitology Department of IEC-SVS/MS, Belém, Pará, Brazil; 3 Oswaldo Cruz Foundation (FIOCRUZ), Rio de Janeiro, Rio de Janeiro, Brazil; 4 Luis D´Écourt Foundation, Belém, Pará, Brazil; 5 Santa Casa Hospital, Belém, Pará, Brazil; Johns Hopkins Bloomberg School of Public Health, United States of America

## Abstract

A total of 179 individuals with acute Chagas disease mainly transmitted by oral source, from Pará and Amapá State, Amazonian, Brazil were included during the period from 1988 to 2005. Blood samples were used to survey peripheral blood for *T. cruzi* hemoparasites by quantitative buffy coat (QBC), indirect xenodiagnosis, blood culture and serology to detection of total IgM and anti-*T. cruzi* IgG antibodies by indirect immunofluorescence assay (IFA) and indirect hemagglutination assay (HA). All assays were performed pre-treatment (0 days) and repeated 35 (±7) and 68 (±6) days after the initiation of treatment with benznidazol and every 6 months while remained seropositive. The endpoint of collection was performed in 2005. Total medium period of follow-up per person was 5.6 years. Also, a blood sample was collected from 72 randomly chosen treated patients to perform polimerase chain reaction (PCR) method. Proportions of subjects with negative or positive serology according to the number of years after treatment were compared. In the endpoint of follow-up we found 47 patients (26.7%) serologically negative, therefore considered cured and 5 (2.7%) exhibited mild cardiac Chagas disease. Other 132 patients had persistent positive serologic tests. The PCR carried out in 72 individuals was positive in 9.8%. Added, there was evidence of therapeutic failure immediately following treatment, as demonstrated by xenodiagnosis and blood culture methods in 2.3% and 3.5% of cases, respectively. There was a strong evidence of antibody clearing in the fourth year after treatment and continuous decrease of antibody titers. Authors suggest that control programs should apply operational researches with new drug interventions four years after the acute phase for those treated patients with persistently positive serology.

## Introduction

During the acute phase of Chagas disease, most patients have a benign prognosis, and the nearly complete remission of symptoms is commonly described to occur between 60 and 90 days, with or without drug intervention. At this stage, specific antibodies help drive the disappearance of parasites from peripheral blood, likely establishing a host-parasite balance. This sustainability depends on the complex immunopathogenic mechanisms that are typical of parasite-host interactions [1], [2].

After the establishment of this dynamic *equilibrium* between the parasite and the host's immune system, three types of disease progression can ensue: development of chronic disease, with detectable lesions in the heart, esophagus and intestines; persistence of anti-parasite antibodies throughout all life of the affected individual combined with incipient cardiac lesions [3]; or, in the indeterminate clinical form, no evidence of established disease with positive serology. Among these possible outcomes, the most serious form of the disease is that which affects heart and digestive tract. It is characterized by low or under levels of parasitemia, persistent levels of anti-*Trypanosoma cruzi* antibodies and serious clinical manifestations in conformity with the location and the extent of injuries that invariably occur decades after initial infection [2].

In endemic areas, there are few records of clinical responses to treatment, even among patients treated during the acute phase [4]. Since 1996, there has been an increasing incidence of orally transmitted Chagas disease outbreaks in Amazon region. It is possible to quickly treat patients who are diagnosed during the acute phase of the disease and accompanied by regular monitoring of treatments [5], [6], [7], [8], [9].

Descriptive follow-up studies of patients treated with Chagas disease hope to identify mediate healing to detect early clinical and/or serological markers of the progression to chronic disease and to establish criteria for earlier indication of retreatment, since the potential of chronicity is smaller than more precocious is the attempt to treat. So, the objective of this study is to describe clinical, serological and parasitological response to treatment with benznidazol in a short and in a medium period of time of persons with acute Chagas disease from Amazon region.

## Methods

### Ethics Statement

The protocol of this work was submitted and approved by the Research Ethical Committee of Evandro Chagas Institute, Ananindeua, Pará State, Brazil. Protocol approval number: 0004/2004. All subjects enrolled provided written informed consent, during 2004 (post acute phase to all of those included before 2004).

### Patients and Study Protocol

This is the first cohort study of Chagas disease realized with treated patients from Amazon region, mainly transmitted by oral route [10]. We included all persons identified in outpatient with acute Chagas disease confirmed by direct and indirect parasitological tests (blood smear or direct examination or Quantitative Buffy Coat QBC, blood culture and xenodiagnosis) and/or a serological test for the acute phase marker (IgM). Patients with infections acquired in Amazon region (Pará and Amapá States), during the period from 1988 to 2005 who agreed to participate in follow-up monitoring were enrolled in the study. Those patients treated prior to 1998 were included based on records from the Chagas disease Laboratory at the Evandro Chagas Institute and were invited to participate in study.

A 20 ml blood sample was collected from patients who agreed to participate. Blood samples were used to survey peripheral *T. cruzi* hemoparasites by QBC, for blood smear, for indirect xenodiagnosis, for blood culture and for serology for the detection of total IgM and anti-*T. cruzi* IgG antibodies by indirect immunofluorescence assay (indirect IFA) and indirect hemagglutination assay (IHA). All of the above assays were performed pre-treatment (0 days) and repeated 35 (±7) and 68 (±6) days after the onset of treatment or during the immediate post-treatment period. With the exception of direct sampling of *T. cruzi,* the other tests were repeated every 6 months after the end of treatment while the patient remained seropositive, with the last collection performed in 2005 (follow-up survey). Monitoring was performed individually with monthly assessments during treatment and every 6 months thereafter. The last assessment was conducted in 2005.

#### Indirect xenodiagnoses

was performed by abdominal compression and collection of sample feces. Samples were placed between a slide and cover slip and observed with an optical microscope (OM). These observations were taken between 30 and 45 days, and again at 60 days after insects feeding. Results were expressed as positive or negative. **Blood culture:** 1 ml blood sample collected from each patient was distributed into five tubes containing Hoff culture medium, prepared as described by Abramo *et al.* (1980) [11]. Treatment failure was recorded if a positive result from one of those parasitological exams was obtained at any time after the completion of treatment (68±6 days).

### Assessment of IgM and Anti- *T. cruzi* IgG Antibodies

For quantitative and qualitative assessments of antibodies, we used indirect IFA and IHA. *T. cruzi* epimastigotes were freeze-dried for immunofluorescence and fixed on slides. After incubation with sera, lyophilized fluorescein-conjugated anti-human IgG antibodies (Fluoline G BIOLAB) or fluorescein-conjugated anti-human IgM antibodies (Fluoline M BIOLAB) were added. Dilutions of 1∶10, 1∶20, 1∶40, 1∶80, 1∶160, 1∶320, 1∶640 and 1∶1280 were used, and titers less than 1∶40 were considered negative. The IHA technique was also used for the qualitative detection of IgG antibodies. To confirm serological clearance of antibodies, the first negative result was followed by two subsequent sample collections at 90-day intervals with repetition of both assays. Individuals with three consecutive negative results by indirect IFA and IHA were considered **serologically cured**.

### Cardiac Evaluation

To evaluate disease progression in those who experienced an acute infection prior to 2002, we reanalyzed recovered electrocardiograms (ECG). We also reexamined existing echocardiograms from the same period. For a subsequent evaluation, patients underwent further electrocardiographic (classic 12-lead ECG) and echocardiographic examination at two reference centers for cardiology: the Luiz Décourt Foundation and Clinical Hospital Gaspar Viana Foundation. The analysis met the criteria stipulated by Brazilian Society of Cardiology [12].

### Radiological Evaluation of the Digestive Tract

Radiological contrast exams of the esophagus and colon were performed on 33 adults who had acute infection for more than four years at the Radiology department of Santa Casa de Misericórdia do Pará Hospital Foundation. For colons radiology patients received information about the procedures and underwent panoramic radiography of the abdomen in an anterior-posterior (AP) position. The contrast agent contained 350 ml of Telebrix (barium sulfate) and 50 ml of saline solution in a final volume of 400 ml. Radiological procedures were performed as follows: a) overview of the abdomen in the posterior-anterior (PA) position; b) right side anterior-posterior position; and c) lateral rectum. After evacuation, patients returned to the examination table for an AP radiograph of the abdomen. For contrast examination of the esophagus, a barium sulfate-based suspension was ingested. With patients in a standing position, three positions were recorded by X-ray: oblique, to observe swallowing disorders; frontal, to observe the gastric mucosa; and in profile, to observe the thoracic esophagus.

### Polymerase Chain Reaction (PCR)

We collected 10 ml of blood from 72 randomly chosen patients among those from follow-up. The collected blood was placed directly into tubes containing a solution of guanidine HCl/0.2 M EDTA and stored at 7°C. Tubes containing blood-guanidine-EDTA were partially immersed in water and boiled for 15 minutes to linearize and release minicircles from the concatenated K-DNA network. A 100-µl aliquot was used for the preparation of DNA. Extraction procedures were performed in duplicate. After deproteinization using phenol-chloroform (1∶1) and chloroform-saturated water, 10% sodium acetate and two volumes of ethanol were added. The mixture was incubated for 15 min on ice and then centrifuged at 12,000 rpm for 15 min. The supernatant was discarded, and the pellet was dried on a hot plate (Multi-block) at 70°C for 5 min. The pellet was resuspended in 50 µl of ultra-pure water, and the DNA was stored at −20°C. PCR was performed according to Britto *et al.* (1995), with 7.5 µl of the extracted DNA in a final volume of 100 µl containing 10 µl of reaction buffer (Buffer II, consisting of 100 mM Tris HCl, pH 8.3 and 500 mM KCl), MgCl_2_ (25 mM MgCl_2_), 100 ng/µl of primers 121 [5′-AAATAATGTACGG(T/G)-GAGATGCATGA-3′and 122 [5′ -CGTTCGATTGGGGTTGGTGTAATATA-3′, which amplified a 330 pb fragment of the conserved micro region of *T. cruzi* kDNA minicircles, 2 µl of dNTPs (10 mM) and 0.75 µl of AmpliTaq Gold (Applied Biosystems) and with modifications performed by the Laboratory of Parasitic Diseases, Department of Tropical Medicine (DMT), FIOCRUZ. The samples were processed and amplified in duplicate. The PCR condition was performed to ensure that all fragment were completely synthesized (95°C for 12′ - 1 cycle/98°C for 1′ - 2 cycles, 64°C for 1′-2 cycles/94°C for 1′/64°C for 1′ - 33 cycles/72°C for 10′ - 1 cycle/4°C for 60′ [13].

As positive and negative controls, DNA was isolated from the blood of confirmed chagasic and non-chagasic patients, respectively. In cases where the PCR result was negative, a second amplification was performed using primers PC03 (forward) (ACACAACTGTGTTCACTAGC) and PC04 (reverse) d(CAACTTCATCCACGTTCACC), which are specific for the human β-globin gene, to determine whether the negative result was due to PCR inhibitors in the samples.

### Treatment Procedures

All patients were treated with benznidazole (Rochagan^®^) (BZ) at a dose of 5 to 7 mg per kg per day for 60 or 90 days, following established medical criteria The treatment was beguine as soon as diagnose was made and this is assured by coordinator of the Cinical Protocol, one of the authors [14].

### Statistical Analysis

For analysis of serological responses the indirect IFA results were available from collected blood samples of follow-up points in time by calculating the geometric mean and standard deviation of IgG anti-*T. cruzi* antibody titers at all collections. Comparisons of IgG titers during follow up were made by Cochran test. Subjects were distributed into groups according to the length of time after onset of treatment (in days and years). The results, expressed as proportions of IFA-negative and IFA-positive patients in these groups, were compared. The Chi-square test was used for this comparison, with a significance level of less than 0.05. The results of the parasitological tests were analyzed from the beginning of treatment and during follow-up in the form of descriptive statistics (frequencies).

For analysis of **clinical conditions,** were considered two points in time: assessments relating to the initiation of treatment (acute phase) and 2005 (end point). We considered the following parameters for this classification: results of serology, electrocardiographic abnormalities compatible with Chagas disease at any phase and/or echocardiographic changes suggestive of chronic Chagas disease. For the analysis of cardiac tests, two blind readers assessed the traces from both tests performed during the acute phase (retrospective) and those made during the follow-up period. Therefore, to provide a cross-sectional classification of the recent clinical condition, a paired comparison was made on a case-by-case basis between results from ECG and echocardiography and from serological and parasitological assays. Co-morbidities of heart disease were also examined individually. Here, we provide a summary of the cardiac analysis that was fully described in an earlier publication [15].

## Results

**Figure 1:**
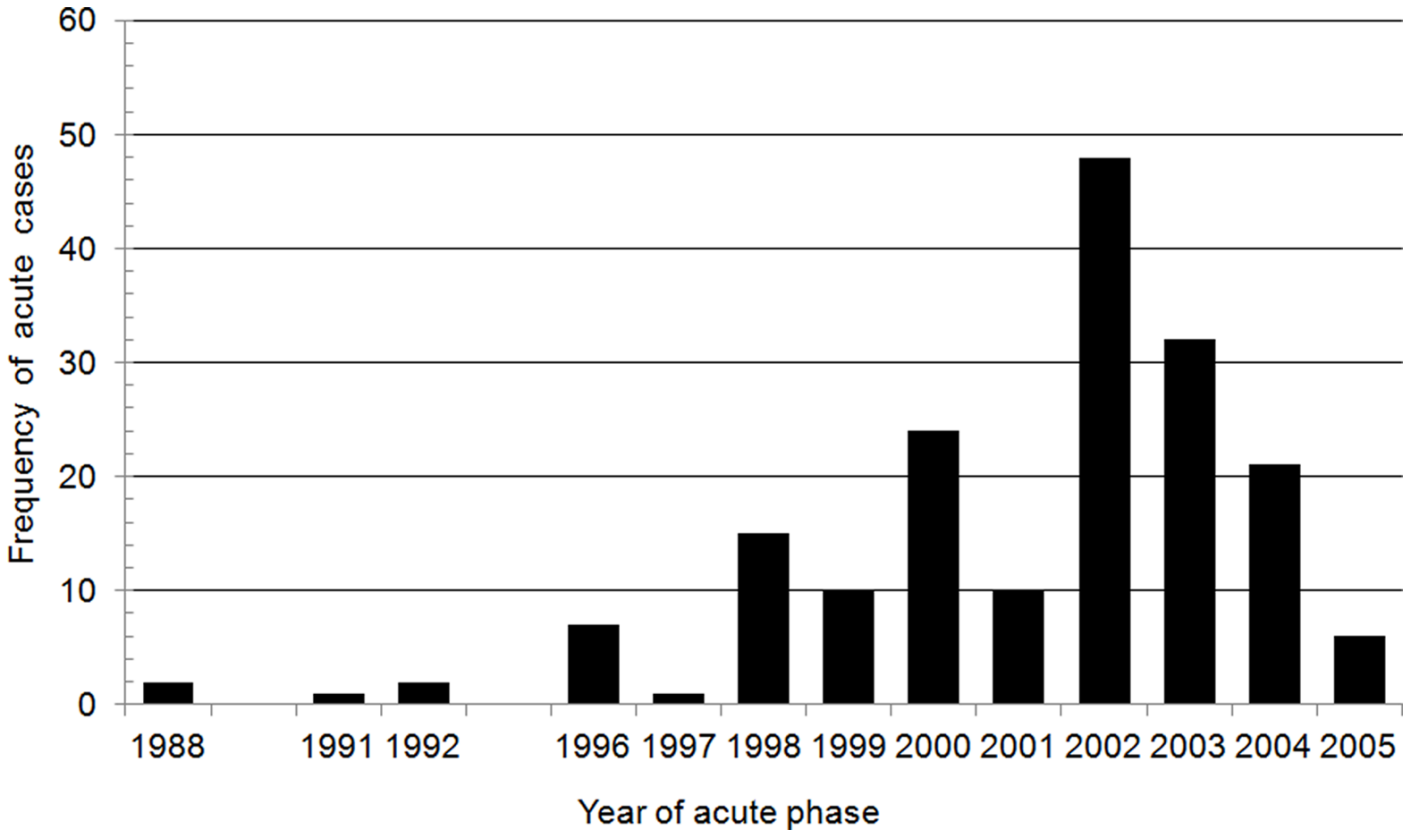
Distribution of acute Chagas disease cases per year of diagnosis.

**Figure 2:**
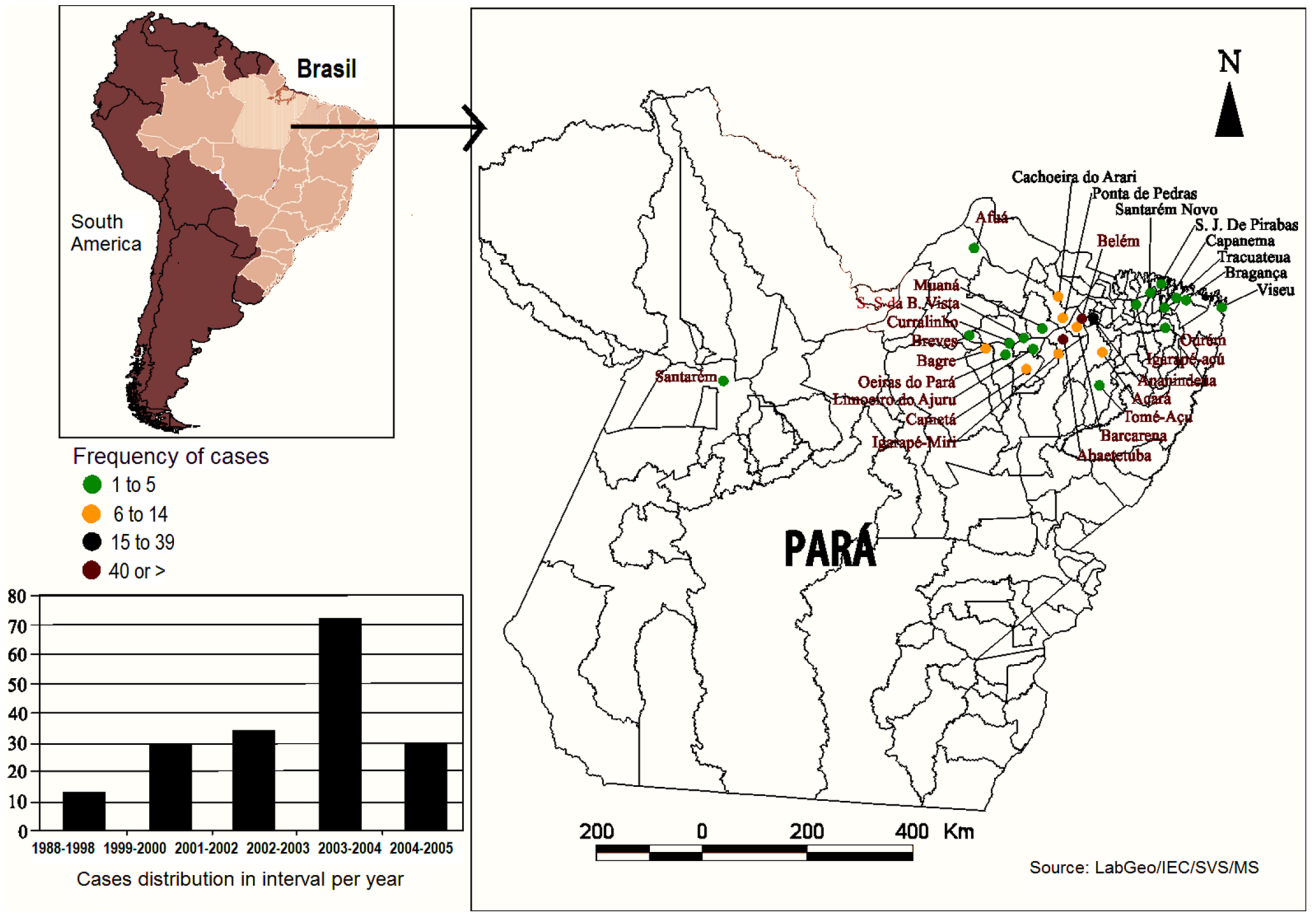
Geographical distribution of studied cases in Amazon region. Adapted from Pinto *et al.,* 2009.

We studied 179 patients between 2 and 72 years of age that had been diagnosed with acute Chagas disease between 1988 and 2005. Patients were included in the study based on their year of acute phase (Figure 1, Data S1, Data S2 and Figure S1). All patients acquired infections in Amazon region distributed in municipalities of Pará and Amapá States (Figure 2). For the study of disease progression after treatment, the average follow-up time was 5.6 years.

### Parasitemia and Serology Follow-up

The percentage of positive tests differed between xenodiagnosis and blood culture. Immediately post-treatment, nine patients had positive results by xenodiagnosis and/or blood culture and were deemed therapeutic failures (Table 1). In the second year after treatment, 100%of the parasitological tests results were negative. One patient had a positive xenodiagnosis 7 years after acute infection concomitant with acute HIV infection, which resulted in reactivation.

**Table 1:** Parasitological indirect exams results during acute phase and follow-up.

Time after treatment	Xenodiagnosis			Blood culture		
	Number of cases	Positive tests	% positive	Number of cases	Positive tests	% positive
Baseline (0 days)	172	110	61.45	172	78	45.34
35±7 days	140	3	2.1%	127	3	2.4%
68±6 days	170	4	2.3%	145	5	3.5%
1 to 1.5 years	151	2	1.3%	172	0	–
2 years	110	0	–	118	0	–
3 to 4 years	86	0	–	60	0	–
5 to 7 years	63	1	1.6%	54	0	–

Titers of anti- *T. cruzi* IgM antibodies were positive in 85.47% (153/179) of cases at baseline (day zero) (see Data S1). From these, 79.08% became negative at the second collection, corresponding to the period between 28 and 42 days after starting treatment. At the end of treatment (between 62 and 74 days after treatment commenced), all patients had negative IgM antibody titers.

**Figure 3:**
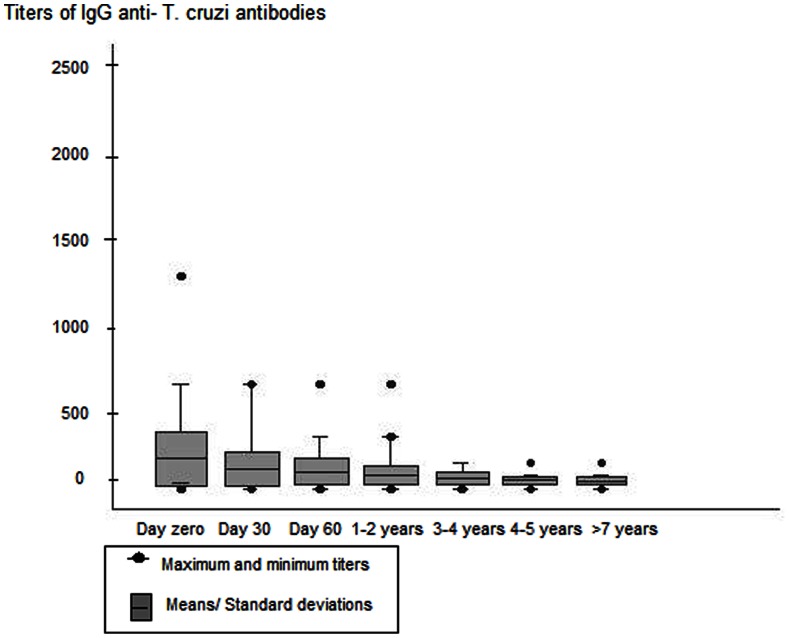
Serological titers of anti-*T. cruzi* IgG antibody during acute phase and follow-up post treatment. Day zero = first day of treatment; Day30 = 30^th^ day of treatment; Day 60 = 60^th^ day of treatment; 1–2 years = 1 to 2 years post treatment; 3–4 years = 3 to 4 years post treatment; 5–6 years = 5 to 6 years post treatment; >7 years = 7 or more years post treatment. Cochran test comparisons: p<0,005.

Titers of anti- *T. cruzi* IgG antibodies were measured during the follow-up and compared for each patient point by point of time, with a significant decrease of titers (p<0,00,5 by Cochran’s Q test) (Figure 3).

Added, we performed comparisons of the proportions of subjects with negative or positive IgG antibodies according to the number of years after treatment. Significant results were obtained when comparing patients treated four years later or less with those treated seven years later or more (p<0.005), demonstrating a tendency toward IgG clearing during this period (Table 2).

**Table 2:** Anti- *T. cruzi* IgG antibody titers measured in treated patients follow-up.

Number of years after treatment	Anti-*T. cruzi* IgG antibody titers					Total positive (%)	Total negative (%)	Total
	1/40	1/80	1/160	1/320	1/1280			
≤1 year*	13	8	2	0	0	23 (76.7)	7 (23.3)	30
2 years	32	22	4	1	0	59 (81.9)	13 (18.1)	72
3 to 4 years*	15	7	4	0	0	26 (76.4)	8 (23.5)	34
5 to 6 years	9	10	1	0	0	20 (66.7)	10 (33.3)	30
≥7 years	2	1	1	0	0	4 (30.8)	9 (69.2)	13
Total	71	48	12	1	0	132 (73.7)	47 (26.3)	179

df = 4 χ^2^ = 12.98 p = 0.011.

* Proportions comparison of treated people (Chi-square or Fisher's test) for different periods after treatment, with significant differences: ≤1 year *versus* ≥7 years = p<0.05, 2 years *versus* ≥7 years = p<0.05, 3–4 years *versus* ≥7 years = p<0.05.

### Cardio-digestive Clinical Follow-up

Among the seronegative (cured) individuals (47), 32 patients (68.1%) had normal ECG in subsequent evaluations. The most important abnormalities observed in this group were diffuse and non diffuse alterations in ventricular repolarization, sinus bradycardia, isolated extrasystoles and left ventricular hypertrophy by arterial hypertension. Echocardiograms were normal in 45 patients (95.7%) from this group.

Among the seropositive cases (not cured) 71.9% (95/132) of the ECG traces were normal. Among the abnormal results (37/132) the mainly electrocardiographic abnormalities are: diffuse and non diffused alterations in ventricular repolarization, SÂQRS deviation, right bundle branch block (RBBB); sinus bradycardia, QRS low voltage, left bundle branch block (LBBB), left-posterior hemiblock and left ventricular hypertrophy (LVH). From this group, five showed electrocardiographic or echocardiographic findings compatible with heart disorders caused by Chagas disease (Table 3).

**Table 3:** Clinical cardiologic summary, IgG anti-*T. cruzi* antibody titers of patients with cardiac abnormalities post treatment.

Year/age of acute infection	Sex	Age atfollow-up*	Follow-up titerIgG anti-*T. cruzi*	Acute phaseECG	Follow-upECG	Acute phaseechocardiogram	Follow-upechocardiogram	Clinicalcondition
2000/15	F	20	40	DAVR, negative T-wave	Blocked right bundle branch.Half-block of posterior leftbundle branch	Normal LVEF = 0.74	Normal. LVEF = 0.71	Cardiacform
2000/17	F	22	80	DAVR, atrioventricular dissociation. Leftaxis deviation	Hemiblock of left bundlebranch. AVR	Mild pericardial effusion.Mild VE dilation. Discrete/moderate diffuse hypokinesia. Mild mitralregurgitation. LVEF = 0.56	Normal. LVEF = 0.62	Cardiacform
2002/11	M	14	40	Low voltage in frontalplane. DAVR	Posterior and inferiorhemiblock	Minimal pericardial effusion. LVEF = 0.66	Mild tricuspid regurgitation LVEF = 0.69	Cardiacform
2003/34	M	36	160	Left atrial overload. Lowvoltage in frontal planesuggesting leftventricular hypertrophy.Right atrialoverload	Left atrial overload. AVR oflower lateral wall	Moderate overall increase.Moderate mitral and tricuspidregurgitation. LVEF = 0.31	Minimal LV increase. Decreasedsystolic performance. Minimalmitral regurgitation LVEF = 0.45	Cardiac form(Myocarditis)
2003/16	M	18	40	Right bundle branchblock. Low voltage QRS.DAVR	T-wave peaked and asymmetric.Sinus bradycardia	Severe pericardialeffusion LVEF = 0.34	Left atrial overload. Diastolicoverload of LV: Dilatedcardiomyopathy. Mild mitralinsufficiency LVEF = 0.48	Cardiac form(Myocarditis)

DAVR = diffuse alterations of ventricular repolarization; LVEF = left ventricular ejection fraction; LV = left ventricle.

* Age at follow-up of recent cardiac assessment.

All results from comparisons of esophageal and intestinal exams were normal.

After analyzing paired results from the serological, parasitological, cardiac and digestive assays we identified four types of recent clinical conditions. Seronegative, i.e., **serological cure,** was observed in 47 (26.3%) patients. In 132 (73.7%) individuals we observed the persistence of anti-*T. cruzi* antibodies (seropositive). From this total, 127 (71%) were characterized as **indeterminate** phase or **seropositive** depending on whether they had or had not undergone digestive assessment, respectively. In five others individuals (2.8%) we found abnormalities consistent with **cardiac form** of Chagas disease (Table 3).

### PCR Results

Six patients (8.3%) had positive results for *T. cruzi* I. All those positives had an acute phase diagnosis less than five years (Table 4).

**Table 4:** Parasitological and serological examinations of cases with positive PCR results.

Year of acute phase	Age	QBC	Bloodsmear	Xeno-diagnosis	Bloodculture	Acute phase titersof antibodies		Follow-up phaseantibodies titers
						IgM	IgG	
1999	75	Neg	Neg	Pos	Neg	160	320	40
2000	28	Pos	NT	Pos	Pos	0	160	40
2002	47	Neg	Neg	Neg	NT	40	80	40
2002	23	Neg	Neg	Pos	Pos	640	0	40
2003	51	Pos	Pos	Pos	Pos	40	80	40
2003	16	Pos	Pos	Neg	Pos	80	160	40

QBC = Quantitative buffy coat.

Pos = Positive; Neg = Negative; NT = Not taken.

Among the six PCR-positive patients, three (50%) received irregular treatments. One patient suspended medication for 15 days due to herpes zoster. The second patient used a dose of 180 mg per day, irregularly. The third patient was febrile for 10 days after beginning treatment, and treatment was interrupted 22 days after dengue fever was diagnosed. None of these patients corresponded to the cases of therapeutic failure detected by xenodiagnosis or blood culture described above. There was no correlation between the type of clinical condition and a positive PCR test (Table 5).

**Table 5:** PCR results and follow-up clinical status post treatment.

Clinical condition	N	PCR-positive (%)	PCR-negative (%)	Total PCR assays
Serologically cured	47	0	9 (100)	9
Indeterminate or seropositive forms	127	6 (9.8)	55 (90.2)	61
Cardiac form	5	0	2 (100)	2
Total	179	6	66	72

## Discussion

Current criteria used to evaluate the success of treatments for Chagas disease are based on technical recommendations from individual experiences recorded in endemic areas. Individuals are considered cured after treatment if results are negative or show a trend towards being negative with a persistent and progressive decline demonstrated by the results from three or more serological tests [14]. This is a criterion very inefficient if we consider the problems related to the serological techniques used, such as discrepancies in the results from treated individuals, persistence of positive serology for long periods among individuals who have used trypanocidal agents and, additionally, the inherent difficulties in using crude antigen techniques [16], [17].

In the cases we investigated IgG antibodies to *T. cruzi* showed a progressive decline, which was most evident between 3 to 5 years after treatment. In addition to serology, we used a series of parasitological assays to assist in evaluating whether the treatment against infection had been failure. The positive cases found among patients during 28 to 42 days of treatment were not considered failures because the patients were still undergoing treatment. However, nine patients who had positive parasitological results from the immediate post-treatment period (52 to 68 days) were considered treatment failures. This finding agrees with those of other authors who have described the effectiveness of the drug and the difficulties in monitoring the cure because these two insensitive methods only identify individuals with the most evident parasitemia. Despite the unsuitable design of the present study for evaluating treatment efficacy, we can interpret these results as an indication of the relative efficacy of the drug used, though the percentage of positive cases immediately after treatment was high for parasitological assays which the sensitivities are notoriously low.

In a placebo-controlled study, xenodiagnosis in 77 patients revealed that there was suppression of chronic parasitemia in 98.1% of cases in the group treated with benznidazole, in 90.4% of the group treated with nifurtimox and in 65.7% of those who received the placebo. The authors acknowledged the difficulty in assessing cure rates through this test because the individuals who used a placebo also had significant "suppression" of parasitemia [18]. Despite the questionable comparability (acute *versus* chronic disease) in the studied cases, we emphasize the validity of xenodiagnosis and blood culture for the short-term follow-up of patients treated during the acute phase because the frequency of positive cases obtained after 54 to 68 days of treatment are comparable with those of other authors. Cançado and Brener (1979) demonstrated the relative efficacy of benznidazol but found a positive xenodiagnosis after treatment in eight out of 20 chronic cases, supporting the theory of the suppressive medication effect, but not curative [19].

In cases of indeterminate Chagas disease form, treatment with benznidazol showed encouraging results from two well-controlled follow-up studies realized in Brazil and Argentina. In Brazil, the treatment with benznidazol produced negative seroconversion in children with this form of the disease. The Argentine study followed patients during four years and demonstrated a good response to benznidazol by clearance of IgG antibodies after treatment as evidence of recovery in 62% of children who used benznidazol, whereas there was no serological improvement in controls who took a placebo [20], [21].

We found 47 individuals patients with negative serology, i.e., serologic cure. Significant differences were observed in the proportions of seronegative individuals acutely infected seven years ago or more and those infected less than four years. However, there were no differences between patients infected at seven years ago or more, and those infected at five or six years ago, which suggests that the period of highest negative results frequency maybe has been occurred up to the fourth year after treatment. The recommendations from endemic areas highlight that, usually, individuals undergo negative seroconversion one to five years after treatment [14]. Our results confirm these observations and also suggest that for the Amazon, this period is shorter and the retesting of patients treated should occur each six months, particularly during the fourth year after an acute infection treatment.

Prognostic interpretations are difficult for 127 patients identified as seropositive without cardiac or digestive changes consistent with Chagas disease. In 33 of these cases, it was possible to undertake a complete evaluation of the cardio-digestive tract, which, according to the criteria used in endemic areas, enabled us to establish that these patients can also be considered with the indeterminate form of the disease. However, critical changes evaluation to special forms of this disease may be evident only after 15 years or longer [22].

In Bambuí (Minas Gerais State, Brazil), 40% of chagasic patients evaluated after the acute phase remained with the indeterminate form for 40 years [1]. In Pains and Iguatama (Minas Gerais), Coura *et al.* (1985) conducted a longitudinal study of patients with the indeterminate form who were free of early heart disease and found that after 10 years, chronic heart disease developed in 38.3% of patients [23].

We found five of those treated patients with established cardiac chronic form of the disease. We assume that the delay in treatment contributed to this result. However, a more aggressive acute phase in four of those five patients seems to have played a decisive role in this outcome. In Bambuí, there was a correlation between progression to chronic heart disease and electrocardiographic changes during the acute phase in patients that were reexamined after 30 years [1].

Autochthonous determinate form of chronic Chagas disease in Amazon has only been reported just in two registers, one of chagasic megacolon [24] and other of dilated cardiomyopathy [25], [26]. These studies indicate an exceptional profile of chronic disease in the region, although such cases have been described since 1969. However, the prevalence rates for chronic phase of the disease in Amazon are nonexistent.

Given the technical difficulties of monitoring and providing diagnostic evidence of a cure in treated patients, we also applied molecular techniques to search for *T. cruzi* antigens in those patients treated for more than two years and six had positive results for *T. cruzi* I. All were seropositive, with low IgG antibody titers. As a technique for assessing treatment success, PCR has shown promising results. In Bolivia, a study 113 children with positive serology or positive QBC and found that 106 of them were also positive by PCR (sensitivity 93.8%). Among the seronegative controls, one positive PCR was detected, but this was attributed to possible sample contamination. Wincker *et al.* (1994) and Britto *et al.* (1995) demonstrated 90% sensitivity of this technique and suggested that PCR is an effective tool to evaluate cure rates, providing a useful adjunct to serological tests [13], [27].

In our study, the proportion of therapeutic failures observed using xenodiagnosis was 2.3%. By blood culture, this failure rate was 3.5%, rising to 9.8% among individuals tested by PCR. Furthermore, positive results detected by these three techniques occurred during different periods after treatment. Failures detected by xenodiagnosis and blood culture were only detected immediately post-treatment, except for one case that was detected after ten years, in which there was a reactivation of Chagas disease due to acute HIV infection (unpublished data). For PCR assays, failures were demonstrated later, with the acute phase varying between 2 and 6 years prior to PCR testing, which suggest that this technique maybe is more sensitive for the evaluation of cure rates. Galvão *et al.* (2003) demonstrated that PCR was 1.6 times more sensitive than serological techniques for detecting therapeutic failures in chronic *T. cruzi* infections treated with benznidazol [28].

The importance of diagnosis and prompt treatment of cases as well as continuous follow-up of patients in public health services by operational clinical protocols of research should be strengthened in the areas of highest risk in Amazon, providing visibility and effectiveness of follow-up methods, in contrast with has occurred in endemic areas in the past.

### Conclusions

The present results show 26.3% of subjects treated in the acute phase demonstrated serologic cure and 2.7% exhibited mild cardiac Chagas disease. Mainly outcome was clinically characterized as chronic, but detection during the acute phase seems to have delayed potential disease progression. There was strong evidence of antibody clearing (serologic cure) in the fourth year after treatment of acute infection. Further, the continuous reduction of antibody titers during follow-up was demonstrated. We suggest that control programs should apply new drug interventions four years after the acute phase for those with persistently positive serology, regardless of titers. Others 73.7% of studied individuals demonstrated positive titers for anti-*T. cruzi* IgG for an average period of 5.6 years after treatment, which may be indicative of parasite persistence. However, decreased titers of IgG antibodies support the evidence of good serological response to drug used in this group. Despite low levels of therapeutic failure immediately after the use of BZ, as demonstrated by xenodiagnosis and blood culture, also occurred by PCR in 9.8% of treated individuals, this do not support evidence of good efficacy of BZ in parasite elimination.

Drug intervention seems to have been decisive in our results; however, an improved assessment can only be made using long-term studies added to use of more sensitive laboratory techniques to evaluate parasite persistence and clinical prognostics.

## Supporting Information

Figure S1 **Results of parasitological and/or serological method performed at baseline diagnosis and compared with each other.**
(TIF)

Data S1 **Baseline descriptive results from parasitological and serological tests performed during acute infection.**
(XLSX)

Data S2 **Baseline results methods comparison with direct parasitological tests (Thick blood film = TBF or Quantitative buffy coat = QBC).**
(XLSX)
