# Supplementary figures and images for: Clinical Follow-Up of Responses to Treatment with Benznidazol in Amazon: A Cohort Study of Acute Chagas Disease
Source: PLoS One. 2013 May 27;8(5):e64450. doi: 10.1371/journal.pone.0064450 (PMC3664625; doi:10.1371/journal.pone.0064450)

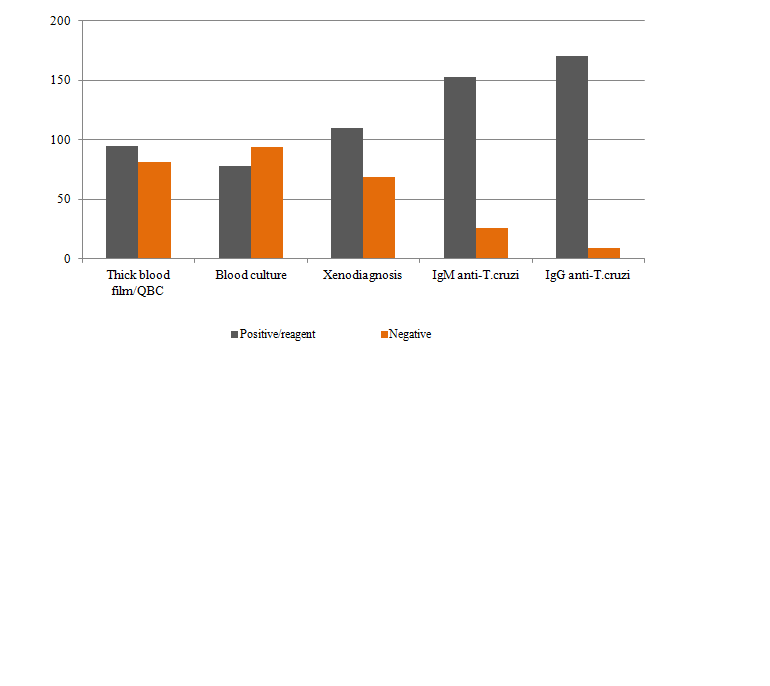

Supplement: Figure S1 — Results of parasitological and/or serological method performed at baseline diagnosis and compared with each other. (TIF) [file pone.0064450.s001.tif]
